# Human TBK1 deficiency: An expanded spectrum from autoinflammation to viral encephalitis

**DOI:** 10.70962/jhi.20250245

**Published:** 2026-02-10

**Authors:** Yemsratch Akalu, Justin Taft, Thomas Berger, Kevin Rostásy, Hormos S. Dafsari, Helena Schönrade, Müşerref Kasap Cüceoğlu, Seza Özen, Albert Faye, Isabelle Melki, Imen Dobroz, Odile Boespflug-Tanguy, Ayça Kiykim, Sezgin Sahin, Eda Tahir Turanli, Ümmüşen Kaya Akca, Jan A.M. van Laar, Philomine A. van Pelt, Iris H.I.M. Hollink, Pallavi Pimpale Chavan, Raju Khubchandani, Ivona Aksentijevich, Dusan Bogunovic

**Affiliations:** 1 Center for Genetic Errors of Immunity, Columbia University, New York, NY, USA; 2 Department of Pediatrics, Columbia University Medical Center, New York, NY, USA; 3 Department of Pediatric Gastroenterology and Rheumatology, Children’s Hospital Datteln, University Witten and Herdecke, Datteln, Germany; 4 Department of Pediatric Neurology, Children’s Hospital Datteln, University Witten and Herdecke, Datteln, Germany; 5 Department of Pediatrics, University of Cologne, Köln, Germany; 6 Max-Planck-Institute for Biology of Ageing, Köln, Germany; 7 Department of Pediatric Rheumatology, Hacettepe University, Ankara, Turkey; 8 Department of General Pediatrics, Pediatric Infectious Disease and Internal Medicine, Robert Debré University Hospital, Assistance Publique-Hôpitaux de Université Paris Cité Paris, Paris, France; 9 Department of General Pediatrics, Paediatric Rheumatology Unit, Armand Trousseau Hospital, Assistance Publique-Hôpitaux de Paris (AP-HP), Sorbonne Université, FHU INFLAMME, Paris, France; 10 Laboratory of Neurogenetics and Neuroinflammation, Imagine Institute, INSERM U1163, Paris, France; 11 Department of Child Neurology, LeukoFrance, Assistance Publique-Hôpitaux de Paris and UMR1141 Université Paris Cité-INSERM, Robert Debré University Hospital, Paris, France; 12 Division of Immunology and Allergy, Department of Pediatrics, Istanbul University- Cerrahpasa, Istanbul, Turkey; 13 Department of Pediatric Rheumatology, Istanbul University-Cerrahpasa, Istanbul, Turkey; 14 Department of Molecular Biology and Genetics, Faculty of Engineering and Natural Sciences, Institute of Natural and Applied Sciences, Acibadem University, Istanbul, Turkey; 15 Department of Pediatric Rheumatology, Akdeniz University, Antalya, Turkey; 16 Departments of Internal Medicine and Immunology, Erasmus University Medical Center, Rotterdam, Netherlands; 17 Department of Rheumatology, Erasmus University Medical Center, Rotterdam, Netherlands; 18 Department of Clinical Genetics, Erasmus University Medical Center, Rotterdam, Netherlands; 19 Department of Pediatric Rheumatology, SRCC Children’s Hospital, Mumbai, India; 20 Department of Pediatrics, Jaslok and Breach Candy Hospitals, Mumbai, India; 21 Inflammatory Disease Section, National Human Genome Research Institute, Bethesda, MD, USA; 22 Department of Pediatrics, Hiroshima University, Hiroshima, Japan

## Abstract

New and long-term follow-up cases of human TBK1 deficiency reveal that patients are vulnerable to severe viral encephalitis in addition to systemic autoinflammation. Findings underscore the dual nature of TBK1 deficiency in combining autoinflammatory disease with impaired antiviral defense.

## Teaser

Akalu et al. report new and long-term follow-up cases of human TBK1 deficiency, revealing that, beyond systemic autoinflammation, affected individuals are also vulnerable to severe viral encephalitis. Their findings highlight the dual nature of TBK1 deficiency—combining autoinflammatory disease with impaired antiviral defense—and emphasize the need for antiviral vigilance in care.

TBK1 is a serine/threonine kinase essential for type I interferon (IFN-I) induction and nuclear factor κ B activation. We previously reported four patients, born to consanguineous families, with homozygous loss-of-function variants in *TBK1* (1). Their phenotype was dominated by systemic autoinflammation, including recurrent fevers, arthritis, vasculitis, seizures, and neuroimaging abnormalities. IFN-I signaling was partially preserved through IκB kinase epsilon (IKKε) compensation, resulting in a hypomorphic antiviral response. This finding helped explain why affected patients presented primarily with autoinflammation rather than overt viral susceptibility.

Since that report, however, six additional patients from four unrelated consanguineous families with TBK1 deficiency have been identified, and longer-term follow-up has become available. Some individuals have now developed severe viral encephalitis or unusually fulminant viral infections, including influenza- and coronavirus disease 2019 (COVID-19)–associated cases with fatal outcomes. These observations expand the clinical spectrum of *TBK1* deficiency and suggest that compensatory antiviral pathways may fail in certain viral contexts, particularly within the central nervous system (CNS).

Here, we report additional patients and longer-term follow-up of previously described cases. Our aim is to highlight the emerging risk of life-threatening viral disease in this disorder and propose antiviral prophylaxis and heightened clinical vigilance as part of standard management (Table 1).

**Table 1.** Clinical features, treatment responses, and outcomes in TBK1-deficient patients

Individual	Family 1		Family 2	Family 3	Family 4			Family 5	Family 6	Family 7
	P1	P2	P3	P4	P5	P6	P7	P8	P9	P10
TBK1 variant	p.W619*	p.W619*	p.Y212D	p.R440*	p. R574Sfs*11	p. R574Sfs*11	p. R574Sfs*11	p.R440*	p.T77_L180del	p.L717Sfs*18
cDNA annotation	c.1857G>A	c.1857G>A	c.634T>G	c.1318C>T	c.1760+4_1760+7del	c.1760+4_1760+7del	c.1760+4_1760+7del	c.1318C>T	c.228+?_540-?del	c.2150_2153del
Transcript reference (GRCh38)	g.64497045 G>A	g.64497045 G>A	g.64474323 T>G	g.64485995 C>T	g. 64496403-ATAAG-A	g. 64496403-ATAAG-A	g. 64496403-ATAAG-A	g.64485995 C>T	g.64463877_64473125del	g.64501340 TTAAC>T
gnomAD (v.4.1.0) frequency	6.21E-07	6.21E-07	Private	1.89E-06	7.94E-07	7.94E-07	7.94E-07	1.89E-06	Private	Private
Predicted consequence/CADD PHRED (GRCh38-v1.7; MSC = 10.854)	39	39	30	36	Predicted LoF (frameshift)	Predicted LoF (frameshift)	Predicted LoF (frameshift)	36	Predicted LoF (deletion of exons 4–5)	Predicted LoF (frameshift)
Age at last evaluation (years)/Sex	34/F	26/M	7/M	11.9/F	2.4/F	1.6/F	3.5/F	4.5/M	19/M	5/F
**Systemic features**										
Recurrent fever	+	+	−	+	+	+	−	+	+	+
Arthritis/joint swelling	+	+	+	+ with contractures	+ oligoarticular, mostly distal/small joints	+ episodic, oligoarticular, small and large joints	+	+	+ polyarthritis, recurrent, self-limited	+
Rash/urticarial or pustular lesions	−	−	+	+ erythematous	+ maculopapular rash	−	+ exanthema	+	+ urticarial rash, pustulosis, neutrophilic dermatosis	+ urticarial rash
Vasculitis	+	+	−	−	+ IgG-vasculitis like	−	−	−	−	−
Lymphadenopathy/organomegaly	−	−	−	+	+	−	−	−	−	−
Aphthous ulcers/mucosal involvement	−	+	−	+	−	−	−	−	+	−
Growth failure/poor growth	+	+	+	+	−	−	−	+	−	−
Other systemic (GI, renal, etc.) issues	+ celiac disease	+ abdominal pain	+ nephrotic syndrome	−	−	−	−	−	+ abdominal pain 1–2×/mo, since 2016, without diarrhea and fever	−
**Neurologic features**										
Developmental delay/regression	+	+	−	+ cognitive and motor retardation	+	+	−	−	+	−
Seizures (type/frequency)	+	+ convulsion (1 year of age), generalized seizure (24 years of age)	+ one episode of seizures with status epilepticus	+ febrile convulsion	+	−	+ several tonic-clonic seizures	+ generalized seizure	+ seizures from 1 mo of age, initially myoclonus and fixed gaze; status epilepticus at 3 and 6 mo; later focal epilepsy with absence seizures	−
Autoimmune/autoinflammatory) encephalitis	−	−	−	−	−	+	−	−	−	−
MRI/CT findings	+ frontal/temporal infarction involving basal ganglia due to cerebral intermediate-vessel vasculitis; hyperdense lesions in basal ganglia and corona radiata	+ multiple white matter lesions in brain and spinal cord	Normal	+ cerebral and white matter atrophy with ventriculomegaly, bilateral thalamic lesions, corpus collosum thinning, and right hippocampal sclerosis; stable chronic encephalomalacic changes	+ brain atrophy, ventriculomegaly/hydrocephalus, lenticular/thalamic lesions, maculopathy	+ brain atrophy, ventriculomegaly, lenticular/thalamic lesions	+ mild diffuse cerebral edema, hyperdense internal veins, and bilateral thalami hypodensities	−	+ hippocampal sclerosis; white matter abnormalities (periventricular, parietal); thalamic atrophy; cavitary sequelae of both lenticular nuclei and the right pulvinar	−
Other	+ stenosis/occlusion of carotid artery; frontal gliosis	+ MS-like disease	​	​	+ microcephaly	+ microcephaly, bilateral central visual impairment, delayed motor development, persistent urinary incontinence	+ urinary incontinence	−	+ microcephaly (z −3); impaired fine motor skills; bilateral maculopathy	−
**Viral encephalitis/infections**										
Viral encephalitis	−	−	−	+ ANEC and ARDS triggered by influenza A/H1N1	+ influenza B	+ influenza B, hemorrhagic encephalitis	−	−	−	−
Other overt viral infections	+ two episodes of severe rhabdomyolysis following viral infection with undefined enterovirus	−	−	−	−	+ CMV (5 mo)	−	+ influenza	+ recurrent HSV-1 (up to 2×/mo, 2010–2018; cheek, labial, oral)	−
Vaccine tolerance/adverse reactions	−	−	−	−	−	−	−	Not reported	−	−
**Treatment and response**										
Corticosteroids	+	+	+	+	+	+	+	+	−	+
Anakinra/Canakinumab	−	+	−	+	−	−	Not reported	−	−	−
Anti-TNF (adalimumab, infliximab, etanercept)	+	+	+	−	+	+	Not reported	+	+	+
DMARDs/immuno-suppressants (MTX, AZA, THAL, COL)	+ MTX, AZA, THAL, COL	−	−	−	−	−	+ MTX	+ MTX	+ COL	+ MTX
Antivirals	−	−	−	+ enfluvir	−	−	+ acyclovir	−	+ acyclovir	−
Treatment efficacy	Was responsive to infliximab	Adverse allergic reaction to infliximab and adalimumab; systemic symptoms partially controlled with anakinra and prednisone	​	Systemic symptoms partially responsive to canakinumab; minimal corticosteroid response; encephalitic episodes refractory	Unresponsive to corticosteroids, partial resolution of fever, arthritis, and skin involvement following treatment with adalimumab	Good initial response to corticosteroids; partial improvement of arthritis and fever under adalimumab therapy; severe neurologic damage following influenza B–associated encephalitis	Prednisolone and methotrexate resolved arthritis	Systemic disease partially controlled with adalimumab; neurologically stable	Partial response to adalimumab with overall improvement, but persistence of brief articular pain episodes and low-grade inflammatory syndrome; neurologic features persist	Arthritis flares under etanercept and acute phase reactants remain elevated, switching to adalimumab
**Outcome**										
Alive	​	+	​	​	​	+	​	+	+	+
Deceased (cause)	+ cause unknown	​	​	+ from influenza A–associated ADEM	+ from influenza B–associated encephalitis	​	+ SARS-CoV-2–asscoiated multi-organ insufficiency	​	​	​
Lost to follow-up	​	​	+	​	​	​	​	​	​	​

LoF, loss of function; GI, gastrointestinal; MRI, magnetic resonance imaging; CT, computed tomography; ANEC, acute necrotizing encephalopathy of childhood; ARDS, acute respiratory distress syndrome; CMV, cytomegalovirus; ADEM, acute disseminated encephalomyelitis; DMARDs, disease-modifying antirheumatic drugs; MTX, methotrexate; AZA, azathioprine; THAL, thalidomide; COL, colchicine; HSV-1, herpes simplex virus type 1; CADD, combined annotation dependent depletion; PHRED, Phred-scaled CADD score; MSC, mutation significance cutoff.

In 2021, we described four patients (P1–P4) with homozygous loss-of-function variants in *TBK1* (P1 and P2: p.W619*; P3: p.Y212D; P4: p.R440*), each presenting in early childhood with systemic autoinflammation (1). The first two patients, adult siblings of Moroccan descent (P1, age 32; P2, age 26, at the time of initial report), developed recurrent fevers, polyarthritis, cutaneous vasculitis, and mild intellectual disability. Both later experienced seizures, with brain imaging showing vasculitic infarcts in P1 and multiple sclerosis (MS)–like demyelinating lesions in P2. Multiple immunosuppressive therapies had limited effect; P1 responded well to tumor necrosis factor (TNF) blockade, whereas P2 experienced severe infusion reactions to TNF inhibitors and remained partially controlled on corticosteroids and anakinra.

The third patient (P3, age 7 at last evaluation, Indian descent) presented with early-onset arthritis and systemic inflammation. He experienced one episode of status epilepticus but otherwise maintained normal cognitive and physical development. His course was complicated by nephrotic syndrome, which responded to corticosteroids. Management has been limited, as the family declined further treatments.

The fourth patient (P4, age 8 at initial evaluation, Turkish origin), first described as part of a broader autoinflammatory cohort, experienced recurrent fevers, aphthous ulcers, lymphadenopathy, and joint pain. She had delayed neurocognitive development, with brain imaging revealing enlarged ventricles, white matter loss, and thalamic atrophy. At the time of the initial report, there was no documented viral infection history, and responses to anakinra were partial.

Follow-up information is now available for some of these individuals. P1 died from an undiagnosed condition, likely infectious, while follow-up information for P2 has not been obtained. P3 remains lost to follow-up, and P4 later developed influenza A–associated acute demyelinating encephalomyelitis and died despite supportive care.

Since the initial publication, we have now been referred two additional patients (P5 and P6), sisters of Turkish ancestry from Germany, with homozygous TBK1 p.R574Sfs*11 variants, who presented with early-onset autoinflammation and a striking course marked by encephalitic episodes. The elder sister (P5, age 2.5) developed intermittent fevers, rash, arthritis, and encephalitis in infancy, from which she initially recovered. She was treated with adalimumab, with good effect on systemic symptoms. The younger sister (P6, age 1) also carried a heterozygous *TNFRSF13B* variant (c.310T>C; p.C104R). Shortly after her second dose of adalimumab, she developed multifocal encephalitis of apparent autoimmune/autoinflammatory origin; infectious workup was negative, and she improved with high-dose corticosteroids, with neurologic deficits gradually regressing. This episode raises the possibility that CNS pathology in TBK1 deficiency may not be exclusively infection triggered and could, in some cases, reflect intrinsic dysregulation of cell death, although this remains to be formally tested. Both children initially remained stable under anti-TNF but, in early 2025, developed fulminant encephalitis triggered by influenza B. P5’s course was rapidly fatal, while P6 required ventilation and sustained severe neurological injury. P6 remains alive but with significant cerebral sequelae. These cases suggest that compensatory IKKε activity supports partial antiviral defense in *TBK1* deficiency but is inadequate in certain viral contexts, leading to fulminant CNS disease and fatal outcomes.

Vulnerability to viral pathogens is not limited to influenza. A separate report described a seventh patient (P7), a 3.5-year-old girl of Turkish descent with homozygous *TBK1* deficiency, who died within days of hospitalization for COVID-19 (2). Despite prior treatment with corticosteroids and methotrexate, she developed seizures, cardiac failure, and multiorgan insufficiency. Genetic testing revealed a homozygous *TBK1* splice-site variant (p.R574Sfs*11) and a homozygous *TNFRSF13B* missense variant (c.310T>C; p.C104R). This patient is a first cousin of P5 and P6, and both sets of parents are consanguineous and related to each other, consistent with inheritance within the same extended family. This finding supports the interpretation that impaired IFN signaling contributed to her unusually severe and lethal course.

Not all patients have shown encephalitic disease. An additional new referral, a Turkish child (P8, age 4.5) with a homozygous TBK1 p.R440* variant, presented at 2 mo of age with fever, rash, and arthritis. He experienced a generalized seizure but has had no further encephalitis or developmental regression. His systemic inflammation remains partially controlled on adalimumab with corticosteroids and methotrexate, but he remains neurologically stable.

A French patient of Turkish descent (P9, age 19) with a homozygous TBK1 p.T77_L180del variant was referred with a phenotype marked by chronic neurologic involvement. He developed convulsive encephalopathy with developmental delay and structural brain abnormalities, including bilateral thalamic lesions, lenticular white matter abnormalities, hippocampal sclerosis, and maculopathy. Systemic features included recurrent febrile episodes without an identified infectious trigger, associated with aphthous ulcers, pharyngitis, skin pustulosis, urticarial rash, abdominal pain, arthritis, and neutrophilic dermatosis. He is currently managed with adalimumab for joint and febrile symptoms and receives valaciclovir for recurrent herpes simplex virus type 1 infections.

A 10th patient (P10, age 5) of Turkish origin with a homozygous TBK1 p.L717Sfs*18 variant presented at 6 mo of age with recurrent episodes of arthritis involving fingers, ankles, and knees. Attacks recurred every 1–2 mo, occasionally accompanied by brief fever, and typically resolved within several days. She was initially diagnosed with juvenile idiopathic arthritis and treated with methotrexate, colchicine, and corticosteroids, with only partial benefit. Since November 2022, she has been maintained on etanercept, which has reduced but not eliminated arthritis flares. There is no history of neurologic involvement, seizures, or serious infection, and her growth and neurodevelopment remain normal.

Taken together, these 10 patients demonstrate both the shared core features of TBK1 deficiency—systemic autoinflammation, arthritis, vasculitis, and variable seizures—and the emerging recognition that viral susceptibility may manifest either as recurrent, manageable infections or, in some cases, as severe, life-threatening encephalitis. Overall, three of 10 patients (33%) died from viral encephalitis, underscoring the potentially fatal nature of this complication. Among individuals with CNS involvement, neuroimaging abnormalities frequently involved deep brain structures (including thalamic and lenticular regions) and were associated with white matter changes, although cohort size and clinical heterogeneity preclude definitive conclusions regarding neuroanatomical vulnerability.

These findings expand our understanding of *TBK1* deficiency. Our initial description emphasized its presentation as a systemic autoinflammatory disease, with recurrent fever, arthritis, vasculitis, and seizures as the dominant clinical features. At that time, impaired IFN-I signaling was recognized, but partial compensation by IKKε was thought to maintain an attenuated but generally adequate antiviral response. Accumulating evidence, however, shows that this compensation is not universally protective. Several patients have now developed fulminant viral disease, most often involving the CNS, and in multiple cases with fatal outcomes.

The occurrence of lethal encephalitis—whether during influenza infection, in the absence of an identified pathogen, or, in one case, in association with COVID-19—broadens the clinical spectrum of this disorder. Importantly, these episodes were not prevented by TNF blockade, which otherwise controlled systemic autoinflammation effectively in most patients. While TNF inhibitors suppress systemic inflammation, they do not address the vulnerability from hypomorphic IFN signaling, which appears insufficient in certain viral contexts, particularly within the CNS.

These observations have several implications for patient care. Clinicians should maintain a low threshold for viral testing and neuroimaging in the setting of new neurological symptoms, even when systemic inflammation appears well controlled. Notably, a recent report published after submission of this manuscript described a child with inherited complete TBK1 deficiency who developed recurrent severe viral infections and fatal post-viral encephalomyelitis, independently supporting the vulnerability to life-threatening viral disease in this disorder (3). Antiviral prophylaxis should be strongly considered, including inactivated influenza and COVID-19 vaccination, as well as prophylactic antivirals where appropriate. At the same time, use of cytokine-targeted immunomodulatory therapies in TBK1-deficient patients should be approached with careful risk-benefit assessment, given the potential to further compromise antiviral immunity. Early intensive care unit–level support may be lifesaving in cases of suspected encephalitis. The potential role of hematopoietic stem cell transplantation remains uncertain but may merit consideration in refractory or life-threatening cases.

In sum, *TBK1* deficiency should be recognized not only as an autoinflammatory syndrome but also as a disorder with critical vulnerability to viral encephalitis. Recognition of this risk is essential for management, and incorporation of antiviral prophylaxis and heightened vigilance into standard care is warranted.
